# Expanding the Role of Women in Vector Control: Case Studies From Madagascar, Rwanda, and Zambia

**DOI:** 10.9745/GHSP-D-22-00508

**Published:** 2023-06-21

**Authors:** Tess Shiras, Meghan Tammaro, Benjamin Johns, Kathryn Stillman, Allison Belemvire, Godfrey Karera, Emmanuel Hakizimana, Timothée Gandaho, Nduka Iwuchukwu, Abigail Donner

**Affiliations:** a Abt Associates, Rockville, MD, USA.; b National Institutes of Health, Bethesda, MD, USA; Formerly of Abt Associates, Rockville, MD, USA.; c U.S. President’s Malaria Initiative, United States Agency for International Development, Washington, DC, USA.; d Abt Associates, Kigali, Rwanda.; e Malaria and Other Parasitic Diseases Division, Rwanda Biomedical Centre, Ministry of Health, Kigali, Rwanda.; f Abt Associates, Antananarivo, Madagascar.; g Abt Associates, Lusaka, Zambia.

## Abstract

**Background:**

Historically, vector control, including entomological monitoring, has been a field dominated by men. Each year, the U.S. President’s Malaria Initiative (PMI) VectorLink project hires 50,000 to 70,000 seasonal workers across the countries in which it works to implement vector control activities, creating an economic opportunity for both men and women. Remaining barriers to women’s employment in vector control include social and cultural norms regarding acceptability of formal employment for women, perceptions that women are not fit to serve as spray operators, and a historical context of male-dominated fields such as entomology.

**Methods:**

We use PMI VectorLink project data from Madagascar, Rwanda, and Zambia for 2019–2021 and key informant interviews with project staff in these countries to examine levels of female employment, effectiveness and efficiency of female versus male malaria spray operators, and strategies to expand the role of women in vector control.

**Results:**

The percentage of female seasonal employees ranges from 25% in Madagascar to 32% in Rwanda and 45% in Zambia. The percentage of women in leadership positions ranges from 32% in Madagascar and Rwanda to 38% in Zambia. Men and women are equally effective and efficient as spray operators. Best practices for recruiting and retaining women in vector control include engaging community leaders in recruitment, implementing affirmative action hiring policies, mentoring women to progress to leadership positions, and ensuring equitable, safe, and attractive workplaces.

**Discussion:**

As vector control programs transition away from donor funding and are increasingly government led, sustaining gains in female empowerment is critical. Country programs should work closely with national, regional, district, and local leaders to demonstrate the importance of hiring women in vector control—including leadership positions—and the impact on female economic empowerment, community well-being, and success of vector control programs.

## Abstract (precis)

We present effective and replicable strategies to integrate women into vector control that provide paid employment opportunities and enhance economic empowerment while strengthening vector control operations.

## Teaser

**Key Findings:**

- Barriers to the employment of women in malaria vector control still exist despite global efforts that have focused on their role and have worked to counteract gender inequity in this area.
- Our analysis fills a gap in the existing literature by demonstrating that men and women have equal ability to serve as spray operators.
- Best practices to recruit and retain women in vector control include engaging community leaders in recruitment, implementing affirmative action hiring policies, mentoring women to progress to leadership positions, and ensuring equitable, safe, and attractive workplaces.

**Key Implications:**

- Country programs should work closely with leaders at all levels to demonstrate the importance of hiring women—including for leadership positions—and the impact on female economic empowerment, community well-being, and the success of vector control programs.
- Community stakeholders, including religious leaders, village leaders, community health workers, women’s groups, and youth groups, can help maintain and expand inclusivity in the vector control workforce.
- Government stakeholders and other implementers should equip mobilization stakeholders to advocate for female engagement in vector control within their communities to advance the goal of gender equality in malaria programming.

## BACKGROUND

The U.S. President’s Malaria Initiative (PMI) VectorLink Project implements indoor residual spraying (IRS), distribution of insecticide-treated nets, entomological monitoring, or a combination of these activities in 25 sub-Saharan African countries to prevent and control malaria, ultimately striving to reduce malaria morbidity and mortality. Each year, PMI VectorLink hires 50,000 to 70,000 seasonal workers across the countries in which it works to implement vector control activities, creating an economic opportunity for both men and women. Building on initiatives in the predecessor PMI Africa Indoor Residual Spraying (AIRS) project, PMI VectorLink takes numerous measures to foster a safe and fair workplace for both men and women and works to increase women’s participation in the vector control workforce, including in supervisory positions. While we recognize that gender is not binary, in this article, we discuss male and female genders—as these are the 2 constructs primarily used in the sub-Saharan Africa region. We use these 2 gender identities to draw comparisons in vector control efficiency of male versus female spray operators.

Historically, vector control, including entomological monitoring, has been a field dominated by men.^1^ A 2018 study on how to increase women’s engagement in malaria vector control at the household, community, and professional levels states that “gender mainstreaming … has not been systematically incorporated into vector control.”^2^ Recently, malaria programs have increasingly placed an explicit focus on the role of women in vector control. For example, the Pan-African Mosquito Control Program has implemented several initiatives to strengthen female leaders’ capacity in the fight against vector-borne diseases and held an event disseminating such work for International Women’s Day in March 2023.^3^ In addition, a gender and malaria community of practice was formed in 2022—currently led by the Centre for Gender Studies and Advocacy at the University of Ghana—that aims to advocate for “the integration of contextualized and intersectional gender-focused approaches for malaria policy, programming, and resourcing at global, regional, national, and local levels in support of malaria eradication and achievement of Sustainable Development Goals.” These efforts generate global attention to and dialogue around the importance of women in vector control and are instrumental in influencing policy and programmatic change.

Recently, malaria programs have increasingly placed an explicit focus on the role of women in vector control.

PMI AIRS was another project that began work to counteract gender inequity in vector control by identifying barriers to women’s engagement in formal vector control employment and operationalizing policies to hire a gender-balanced workforce. These policies included adapting physical work sites to ensure privacy for women in dressing areas and bathrooms, providing menstrual hygiene products for female seasonal workers, guaranteeing job security and enhancing job safety for pregnant women, enforcing strict anti-harassment policies, and encouraging female staff to apply for supervisory positions. In the PMI AIRS project, female employment increased from 23% in 2012 to 29% in 2015, and the percentage of women in supervisory roles grew from 17% in 2012 to 46% in 2015.^4^

Formal employment increases women’s economic empowerment, which can affect social mobility, autonomous decision-making, and agency—defined as control over social and material resources and the ability to pursue what one values.^5^^,^^6^ Employment provides women with their own finances and, depending on how they are paid, direct control over those finances. Increased women’s economic empowerment is also associated with improved maternal and child health outcomes, reduction in gender disparities, and more efficient allocation of household resources.^7^^–^^9^ A study in Kenya and Indonesia found that employing women in vector control could lead to more sustainable programs and have economic and social empowerment benefits for spray operators—including acting as role models for the next generation.^2^

Despite these benefits, barriers to the employment of women in vector control still exist. In many countries, social and cultural norms are a barrier to women’s participation in the formal job market.^10^ Deep-seated cultural beliefs—such as the notion that it is inappropriate for women to have formal jobs, as their primary obligation is child and household caretaking—inhibit barriers to employment entry. Specifically, the spray operator position requires handling perceived heavy equipment and can include travel, including overnight travel, which can be deemed inappropriate for women.^11^ There is scant literature demonstrating women’s ability to effectively execute this job. Another barrier to employing women in vector control is limited awareness of career opportunities.^11^ However, 1 study found that once women were employed in vector control, they were 6 times more likely to be encouraged to pursue leadership positions than their male counterparts, suggesting that professional development is not a barrier for women once they are working in the sector.^11^

The PMI AIRS project developed an operational foundation for formally employing women in vector control. Using 3 country case studies—Madagascar, Rwanda, and Zambia—we examine strategies that PMI VectorLink has taken to expand the role of women in vector control, including as spray operators, entomologists, and supervisors. To fill a gap in the literature and overcome barriers to hiring female spray operators, this article also provides data on the effectiveness and efficiency of spray operators by sex.

We use country studies on Madagascar, Rwanda, and Zambia to examine strategies PMI VectorLink has taken to expand the role of women in vector control.

## PMI VECTORLINK GENDER EQUITY STRATEGIES

The PMI VectorLink Project is the largest global implementer of IRS. The project began in 2017 and currently works in 25 countries to implement IRS, insecticide-treated net distribution, and entomological monitoring. PMI VectorLink implements policies developed under PMI AIRS to create a respectful, safe, and fair workplace for both men and women.^4^

### Privacy at Work

PMI VectorLink has updated operational sites so that there are separate changing areas, bathrooms, and showers for men and women, as spray operators must shower after spraying. These facilities must lock, have doors from the ground, and be high enough to ensure privacy. Trash cans must be provided for menstrual hygiene products, and showers must have proper drainage so all residual water drains, which is key when women are menstruating.

### Safety at Work

There is zero tolerance for harassment, including sexual harassment. All seasonal employees undergo gender and sexual harassment awareness training. Sexual harassment guidelines with a local contact number for any misconduct are posted at each operational site in all relevant local languages. All female spray operators work with a female buddy to accommodate social norms in countries that discourage a woman from working alone with a group of men. When spray teams require overnight accommodations, men and women always stay in separate, secure accommodations.

### Job Security During Pregnancy

Insecticide exposure is unsafe for pregnant and lactating women. In alignment with World Health Organization guidance, PMI VectorLink requires all female employees in positions with possible insecticide exposure to take a pregnancy test every 30 days during the spray campaign. Test results are given confidentially, and any woman who is pregnant is provided with another position without insecticide exposure at the same pay grade.

### Professional Development

PMI VectorLink strives to promote talented women into leadership positions. To retain talent, the project encourages previous female employees to apply for leadership positions in the next year’s spray campaign. PMI VectorLink implements an affirmative action program to give priority to qualified female applicants for leadership positions. Some country programs have instituted a mentoring program to help female employees to develop their leadership skills.

### Equity-Focused Procurement

PMI VectorLink provides menstrual hygiene products to all female seasonal employees. In addition, the project procures personal protective equipment in a variety of sizes to ensure a safe and comfortable fit for people of all sizes. In some countries, PMI VectorLink procures 2-piece protective gear with a long skirt instead of coveralls, as this option can be more comfortable for some women.

### Mobile Money Payments

Payments to all seasonal workers are made through mobile money applications, which provide safe and reliable payments. Mobile-based payments have particular advantages for women, as they help to increase financial autonomy and decision-making authority for employees. Women have direct access to electronic funds, which hypothetically allows them to have more control over their money, as employees can use their phones to manage their finances, including money deposits, transfers, and cash-outs.

## METHODS

We purposively selected Madagascar, Rwanda, and Zambia as case study countries because they represent varying levels of female employment on PMI VectorLink (Rwanda: high; Zambia: midlevel; and Madagascar: lowest). We use mixed methods to answer the following research questions:

- What percentage of seasonal workers were women in Madagascar, Rwanda, and Zambia by year and employment cadre? What was the associated economic impact of this employment?
- How efficient and effective were female spray operators compared to males in Madagascar, Rwanda, and Zambia?
- What are the successful strategies for and challenges related to hiring women in vector control in Madagascar, Rwanda, and Zambia?

To answer the first research question, we used PMI VectorLink project data from 2019 to 2021. Project data documents the number of seasonal employees, disaggregated by sex. We examine data on the overall number of seasonal employees, spray operators, and seasonal supervisors in 2019, 2020, and 2021. We also examine the percentage of female employees working in entomology for 2020 and 2021. Entomology data disaggregated by sex is not available from 2019.

To analyze the second research question on spray operator efficiency and effectiveness, we examined several program indicators by sex in 2020 and 2021. Metrics include: (1) average number of days worked per spray operator; (2) average number of structures found per spray operator per day; (3) average number of structures sprayed per spray operator per day; and (4) average percentage of structures found that are sprayed. We used t-tests with robust standard errors to assess if results were statistically significant (*P*<.05) between men versus women in each country and pooled across 3 countries. Pooled results use a weighted average so that countries with more spray operators represent more of the average. We controlled for the subdistrict of spray operation, as geographic location may affect spray operator success. These programmatic data were not available from 2019, so this analysis is limited to 2020 and 2021.

To answer the third research question, the authors conducted key informant interviews with full-time PMI VectorLink colleagues in Madagascar, Rwanda, and Zambia who oversee gender mainstreaming in their country teams. Two informants were interviewed from each country—the chief of party, who oversees all technical implementation and operations, and the equity focal point, who is responsible for implementing gender-mainstreaming policies, including female recruitment—totaling 6 key informants. The purpose of these interviews was to better understand each country’s strategies for hiring women in vector control, successes and challenges in these efforts, and the perceived impact of hiring women on the program, on employees, and on communities. We used a semistructured interview guide to explore these themes and help contextualize changes in female recruitment levels from 2019 to 2021. The interviews explored challenges in recruitment, the role of the COVID-19 pandemic in hiring women, and future plans for increasing female recruitment. All interviews were recorded, and a notetaker took detailed, thematic notes (not verbatim). Two researchers reviewed interview notes and synthesized findings to distill key themes and help contextualize quantitative data.

## RESULTS

### Amount of Women Employed in Vector Control

The percentage of women employed in vector control varies in each country and by type of employee. In some cases, the percentage of female employees decreases while the absolute number increases due to an overall increase in the size of the country’s vector control program (e.g., Rwanda from 2019 to 2020). Results from 2019 to 2021 for the 3 countries are summarized in Table 1.

**TABLE 1.** Women Employed in Seasonal Vector Control in Madagascar, Rwanda, and Zambia, 2019–2021

**Employee Category**	**Year**	**Madagascar, No. (%)**	**Rwanda, No. (%)**	**Zambia, No. (%) **
All seasonal employees	2019	987 (35.0)	1,453 (39.5)	2,175 (24.0)
	2020	263 (21.2)	1,894 (28.5)	5,663 (41.9)
	2021	314 (24.7)	2,177 (32.3)	9,511 (45.2)
Spray operators	2019	81 (9.8)	891 (52.0)	517 (35.2)
	2020	83 (11.8)	863 (49.6)	780 (44.0)
	2021	111 (15.5)	973 (52.1)	814 (40.7)
Supervisors	2019	73 (30.0)	303 (48.0)	22 (25.0)
	2020	55 (28.9)	334 (48.6)	269 (37.0)
	2021	65 (32.2)	351 (48.7)	300 (38.0)
Entomology employees	2020	77 (41.2)	64 (47.8)	41 (11.4)
	2021	99 (36.0)	68 (47.2)	39 (12.2)

#### All Seasonal Employees

While the percentage of all female seasonal employees decreased moderately in Madagascar and Rwanda from 2019 to 2021, it increased substantially in Zambia, from 24% to 45%. The 3 countries each represent a different benchmark in female representation: one-quarter of seasonal employees are female in Madagascar, one-third in Rwanda, and just less than one-half in Zambia. Additional information regarding each country’s recruitment strategies is included in the next section.

From 2019 to 2021, the percentage of all female seasonal employees decreased moderately in Madagascar and Rwanda but increased substantially in Zambia, from 24% to 45%.

#### Spray Operators

Rwanda has achieved a gender-balanced spray operator workforce, with 52% female spray operators. In Zambia, 41% of spray operators are female, which represents a 6 percentage point increase since 2019 at 35%. The situation is quite different in Madagascar, where just 16% of spray operators are women. This has increased by more than 50% since 2019, when just 10% of spray operators were women.

#### Supervisors

In Madagascar and Zambia, approximately one-third of supervisors are female (Madagascar: 32%; Zambia: 38%). In Madagascar, this percentage has remained fairly stable since 2019. Again, the percentage in Zambia increased from 25% in 2019 to 38% in 2021. In Rwanda, the percentage of female supervisors is much higher at 49%.

#### Entomology Employees

In Madagascar, over one-third (36%) of entomology employees are women. This statistic is even higher in Rwanda at nearly half (47%). It is the lowest in Zambia at 12% and is the 1 employee category for which Zambia has less than one-third female representation.

### Economic Impact

Across the 3 countries, PMI VectorLink hired 12,002 female employees in 2021, providing women with paid employment opportunities within their communities. This included 1,898 female spray operators and 2,542 female supervisors. The daily spray operator wage in 2021 ranged from US$2.93 in Zambia to US$4.47 in Madagascar and US$5.49 in Rwanda. On average, female spray operators in these 3 countries earned US$100.25 for the 20–30 day spray campaign. This short-term seasonal employment provides an opportunity for women to receive income when many may not be able to commit to full-time employment given household and family responsibilities.

### Spray Operator Efficiency and Effectiveness

On average, across the 3 countries in both 2020 and 2021, men performed almost equally to women on 2 of the 4 indicators assessed (Table 2). Male spray operators found slightly more structures per day (2020: 13.4 vs. 13.2, *P*=.001; 2021: 12.9 vs. 12.7, *P*<.001) and sprayed slightly more structures per day (2020: 13.0 vs. 12.7, *P*=.006; 2021: 12.5 vs. 12.3, *P*<.001). In 2020, there was no significant difference between male and female spray operators in the average number of days worked nor in the average percentage of identified structures that were sprayed. In 2021, female spray operators were likely to work slightly more days than their male counterparts (21.6 vs. 21.4, *P*=.018).

**TABLE 2.** Spray Operator Efficiency and Effectiveness on Average Across Madagascar, Rwanda, and Zambia, 2020 and 2021

**Indicator**	**2020**			**2021**		
	**Women**	**Men**	***P* Value** ^a^	**Women**	**Men**	***P* Value**^a^
Average number of days spray operator worked	22.4	22.4	.72	21.6	21.4	.02
Average number of structures found per spray operator	13.2	13.4	<.01	12.7	12.9	<.01
Average number of structures sprayed per spray operator per day	12.7	13.0	<.01	12.3	12.5	<.01
Average percentage of structures found that are sprayed per spray operator, %	96.3	96.5	.09	96.45	96.52	.52

^a^ Significant at *P*<.05.

On average, across the 3 countries in both 2020 and 2021, men performed almost equally to women on 2 of the 4 indicators assessed.

In both years, differences were not significant for any of the 4 indicators in Madagascar, demonstrating that male and female spray operators are equally effective. The same holds true for Rwanda in 2021. In 2020, male spray operators in Rwanda performed almost equally to female spray operators for the average number of structures identified (11.2 vs. 11.1, *P*=.023) and sprayed (11.1 vs. 11.0, *P*=.042). In Zambia, men and women also performed nearly equally for number of structures identified (2020: 14.7 vs. 14.5, *P*=.02; 2021: 14.5 vs. 14.1, *P*<.001) and sprayed (2020: 14.0 vs. 13.7, *P*=.004; 2021: 13.7 vs. 13.4, *P*<.001). In 2020, male and female spray operators were nearly equally successful in spraying identified structures (95.3% vs. 94.8%, *P*=.043). In 2021, female spray operators in Zambia worked more days than male spray operators on average (26.3 vs. 25.9, *P*<.040).

### Strategies to Employ Women in Vector Control

The 6 key informants (chief of party and equity focal point in each country) to whom the authors reached out all participated in an interview. PMI VectorLink staff in Madagascar, Rwanda, and Zambia shared successful strategies and challenges in recruiting and retaining female employees and shed light on the various patterns documented in the previous section.

#### Madagascar

The PMI Madagascar team aims to have women as 35% of their seasonal workers. This goal was achieved in 2019, but the percentage decreased to 21% and 25% in 2020 and 2021, respectively. The higher percentage of female workers in 2019 is attributed to a special household enumeration activity that occurred that year. The team recruited these household enumerators from a pool of existing community health workers (CHWs), who are primarily women. The PMI Madagascar team confirmed that the COVID-19 pandemic did not have a negative implication on the number of women engaged in vector control employment.

The Madagascar team works with village chiefs and CHWs to help recruit women into vector control positions. The PMI VectorLink team holds informational sessions with these key community leaders and asks them to proactively recruit and encourage women to apply to all seasonal employee positions, highlighting that women have a right to equal employment opportunities. When VectorLink Madagascar holds larger community mobilization events to prepare for IRS, they also advertise opportunities for women and encourage them to apply. All job descriptions also specifically state that women are encouraged to apply. These efforts help to transform social norms around women’s engagement in the formal labor market and normalize their active participation.

The Madagascar team has worked to transform social norms around women’s engagement in the formal labor market and normalize their active participation.

However, in some districts where VectorLink Madagascar works, the presence of stricter gender norms makes it more difficult for women to engage in paid economic opportunities and can pose security concerns for women. VectorLink Madagascar ensures that employees work in groups rather than individually to promote safety. In addition, the VectorLink team noted that consistent and comprehensive training on sexual harassment has helped to create a safe work environment and attract women to the VectorLink team. These strategies are gender sensitive, working within the existing norms framework. Transforming these deep-seated cultural norms to work toward perceptions of gender equality in the workforce would require much more intensive and comprehensive social and behavior change.

The Madagascar team has put a particular emphasis on hiring and promoting women for supervisory positions, achieving one-third female supervisors. While reviewing applications, Madagascar VectorLink prioritizes female applicants when male and female applicants are equally qualified. The team reported that although initially recruiting women into vector control is a challenge, retaining and promoting women to supervisory positions is less challenging, which indicates that the PMI VectorLink measures to create an equitable workplace have been successful. The Madagascar team also shared that some male employees expressed dissatisfaction with having a female supervisor. The VectorLink team responded to this concern swiftly, using equity and rights-based language to explain that women are equally skilled and deserving to be supervisors and that gender equity is exemplified in all aspects of VectorLink, including supervision.

Working in remote areas is 1 challenge the Madagascar team has confronted in recruiting female spray operators, with just 16% female spray operators in 2021. For example, spray operators must walk 6–8 hours in a day to reach extremely rural villages in Iakora and Ihosy that are difficult to access via bike or motorcycle, and security can be a concern in these areas. These factors may dissuade women from applying to these jobs, particularly if they have families to care for and lack alternate childcare. The team plans to focus on increasing female representation in spray operators by hiring directly from remote communities and retaining female employees from previous years. Entomologists also face challenges with respect to long periods away from home. They typically work in the field for 20 days per month. Despite this challenge, the Madagascar team has achieved high female representation among entomologists in recent years—41% in 2020 and 36% in 2021—by largely recruiting from universities and retaining female entomologists over time.

#### Rwanda

The PMI Rwanda team highlighted that the Government of Rwanda’s commitment to prioritizing gender equality and social inclusion is key to the project’s success in female engagement in vector control. There is a legal framework in place to promote women’s equal rights and employment opportunities. The Rwandan constitution mandates that women represent at least 30% of all government decision-making positions across levels. A Gender Monitoring Office was created to oversee gender integration across government levels. Rwanda has the highest share of female parliamentarians in the world at 61%.^12^

In Rwanda, the government’s commitment to gender equality and social inclusion is key to the project’s success in female engagement in vector control.

VectorLink Rwanda works closely with government partners to mainstream gender, including through employment opportunities. The Government of Rwanda determined that spray operators should be recruited from CHWs since they already play a vital role in delivering health services and information to their communities and trust has already been established. In Rwanda, the CHW workforce is gender balanced, so recruiting from this cadre has resulted in gender equity for spray operators on the VectorLink project, as well. Spray operator recruitment is a transparent process in Rwanda. All CHWs who work in the relevant districts are called to a meeting so that everyone has an equal opportunity to apply to work on IRS campaigns. The Rwanda team noted that workplace factors, such as distribution of sanitary pads and separate and secure bathrooms, showers, and changing facilities, have facilitated female engagement and retention in malaria vector control. The VectorLink team confirmed there that the COVID-19 pandemic did not have a negative effect on recruiting or retaining female employees.

For supervisor recruitment, the Rwanda VectorLink team holds microplanning meetings with the Malaria and Other Parasitic Diseases Division of Rwanda Biomedical Centre, CHWs, and health facility representatives to advertise opportunities and encourage women to apply. Rwanda achieved 32% female supervisors in the 2021 spray campaign. The team strives to retain supervisors and promote high-performing spray operators to team lead positions in subsequent years.

Some seasonal employee positions are not selected by the VectorLink team but by the Government of Rwanda. For example, the government has determined that community mobilizer positions will be assigned to the village lead and village security lead in each community. These are elected positions, and they are heavily male dominated. Although 30% female participation is expected based on the national legal mandate, this does not always occur. The VectorLink Rwanda team had many more community mobilizers included in their 2020 and 2021 spray campaigns compared with 2019, which explains the overall decrease in the proportion of female seasonal workers from 2019 to 2020 and 2021. Entomology monitoring is another area where the government, rather than VectorLink, oversees hiring seasonal staff.

The VectorLink Rwanda team reports that hiring women on the project has positively impacted female employees’ social and economic welfare. For example, a female employee who was hired in 2019 as a spray operator and has since been promoted to a pump technician shared that the living standards for her family have improved since she joined VectorLink.

*Before IRS, it was impossible to spend 5 days without seeing someone who was suffering [from] malaria. I have seen how deadly malaria was to my household. What motivates me about IRS is the protection it gives to the community against malaria and an income that I normally earn during the spray campaign, which has improved living standards for my family.* —Female pump technician from Ngoma District, Rwanda

She has been able to afford annual health insurance for her entire family and has increased spending on school materials for her children. In addition, she noted that she feels proud to be one of the few female pump technicians and contribute to the fight against malaria for her community.

#### Zambia

The Zambia VectorLink team works with provincial and district officers to recruit seasonal employees. These officers regularly interact at the community level, so gender mainstreaming messages are well received. As part of recruitment, officers communicate that all employees receive gender sensitivity training as part of their regular training. This helps to convey the importance of gender mainstreaming to the VectorLink team and attract female applicants. In addition, during community mobilization efforts, the Zambia team uses graphics that depict both male and female spray operators to help break down perceptions that this is a male-dominated role.

The Zambia team saw a large increase in female participation across nearly all employee categories from 2019 to 2020, which was attributed to implementation of a recruitment protocol designed to boost women’s participation. The team deployed a survey among district coordinators in 2020 asking for actionable recommendations to increase female participation. In collaboration with the National Malaria Control Programme, the Zambia VectorLink team identified common recommendations and created a revised recruitment protocol, which includes the following strategies:

- **Increase engagement of community leadership**. In addition to working with provincial and district officers, Zambia VectorLink works with village leaders, traditional leaders, and church leaders to encourage their female community members to apply for seasonal positions.
- **Consistently check in with individuals overseeing recruitment.** This allows for regular status updates and the opportunity to make any midcourse changes if recruitment progress is not on track.
- **Communicate anti-harassment policy and other workplace policies**. In all meetings from the national to the community level, the Zambia team reiterates its zero-tolerance policy for sexual harassment and guidelines for safely reporting any incidents. In addition, they share workplace features, such as distribution of sanitary pads and paid menstrual leave—per Zambian law, separate and private changing and shower facilities, and job protection with equal salary if a woman becomes pregnant.
- **Proactively encourage high-performing previous employees to apply for supervisory positions**. This helps to retain talent and encourage professional development.

In Zambia, a new recruitment protocol boosted women’s participation across nearly all employee categories from 2019 to 2020.

Implementation of this recruitment protocol resulted in 42% and 45% female seasonal employees in 2020 and 2021, respectively. The Zambia team has a goal of a minimum of 40% female employees each year, including for supervisors and in entomology. Zambia has had difficulties recruiting female entomology employees, with just 12% women in 2021. Entomology employees include: (1) environmental health technicians, who are hired by the government and are technical positions; and (2) community entomology employees, who are more likely to be female than environmental health technicians. However, many of the community entomology employees conduct human landing catches to monitor mosquito behavior, which involves working through the night. There are gender norms that discourage women from working through the night, so this position is less attractive to female candidates.

The Zambia team reported that hiring women in vector control has a remarkable impact both on women’s own livelihoods and on the success of IRS campaigns. IRS is conducted in many small villages where there are few or no paid employment opportunities for women. Female spray operators have reported that they feel proud to work on malaria prevention in their communities.

*I am now motivated. There are so many malaria cases and death. I know how mothers and children suffer from malaria. So, it gives me pride and joy to be part of this campaign and to know I’m part of the people fighting against malaria.* —Female spray operator in Zambia

The Zambia team noted that female spray operators are also helpful in increasing IRS acceptance. IRS occurs during the day, when many men are away at work, and often women are the ones to prepare their homes for IRS and provide consent for spraying. Having women represented in the spray workforce is helpful in facilitating community acceptance. In addition, women in supervisory positions act as role models to other girls and women in their community as well as to female spray operators, showcasing that women can be leaders for change.

## DISCUSSION

Our analysis fills a critical gap in the existing literature by providing critical best practices for successfully hiring women in paid vector control employment opportunities, including leadership positions, and demonstrating that men and women have equal ability to serve as spray operators. As vector control programs transition away from donor funding and are increasingly government led, sustaining gains in female engagement and empowerment is critical.

Findings from Madagascar, Rwanda, and Zambia show that men and women are equally efficient and effective as spray operators. Any significant differences between male and female spray operators were on the magnitude of 0.2 to 0.3 households identified or sprayed per day, on average across the 3 countries. While the differences may be statistically significant, they are not operationally significant. These data combat misperceptions that women are less able to perform spray operator duties and are equally able to identify and spray households. Given the systemic marginalization of women from paid employment across many settings where PMI VectorLink operates, the significant economic benefits of increased women’s employment and the impact of this employment on shifting gender norms outweigh the small observed differences in structures found and structures sprayed. Formal employment opportunities and financial compensation for women in the health system are critically important, particularly in the context of the large number of unpaid or underpaid women who contribute to their country’s health.^13^ Additionally, the observed differences cannot be generalized to all IRS settings or to other vector control activities, such as insecticide-treated net distribution or entomology work. Additional research on this topic is warranted in other vector control activities and other geographic settings outside of sub-Saharan Africa.

Our data combat misperceptions that women are less able to perform spray operator duties and are equally able to identify and spray households.

### Best Practices

Best practices from Madagascar, Rwanda, and Zambia for increasing and retaining female employment in vector control can be emulated in other countries. Such practices include:

- Working with community-level leaders (including through nongovernmental organizations and community-based organizations) and empowering them to serve as champions for female employment in vector control.
- Implementing an affirmative action program to give priority to qualified female applicants.
- Mentoring female employees to help them progress to leadership positions and encouraging high-performing female employees to apply for leadership positions.
- Creating an equitable, safe, and attractive workplace through policies such as appropriately sized personal protective equipment, distribution of sanitary pads, separate and private bathroom facilities, and clear and enforced anti-harassment policies.

In addition to these practices, adapting outreach tactics to appeal to women or overcome barriers to female engagement is an important strategy. For Zambia’s 2022 spray campaign, the team plans to focus its gender-mainstreaming efforts on districts where female participation is lower than average. The team will query district officers to brainstorm factors that could contribute to these lower numbers and then implement tailored communication techniques to increase female recruitment. Community-based or civil society organizations that are already well connected with community members are also important resources to leverage in these efforts.

The Rwanda case study demonstrates the impact that national-level policy has on female empowerment. The mandate that women make up 30% of all government decision-making positions has normalized women in leadership positions and helped to mainstream gender equality. To this end, the VectorLink Rwanda team has faced few challenges in encouraging women to participate in vector control employment and has consistently achieved approximately one-third or more women across employment categories.

While Madagascar and Rwanda have achieved approximately one-third and one-half, respectively, of their entomology employees as women, this is an area that continues to be viewed as a male-dominated field in some locations. Best practices to engage women in entomology include hiring local women to serve as mosquito collectors and using a buddy system to ensure women are not working long hours alone or with a man if there are security concerns or other cultural considerations. In addition, the entomology field should prioritize training and mentoring women, beginning at the university level. It is also important for entomology institutions to conduct gender-balanced outreach to proactively encourage more women to pursue formal training, internships, and careers in entomology. Teams can also focus on training more women as technicians at sentinel sites regardless of previous entomology experience.

Effective IRS depends on household acceptance to ensure high coverage. In many communities where PMI VectorLink operates, men disproportionately have household decision-making authority. However, IRS typically occurs during the weekday when many men are away from home, giving women this decision-making authority. Studies have attributed household IRS refusal to several gendered factors, primarily related to women’s multiplicity of household roles such as cooking, cleaning, health care responsibilities, and childcare. For example, a woman may feel uncomfortable with a male spray operator entering her home, be concerned about insecticide, be bothered by the extra burden of removing household items in preparation for spraying, or feel as though educational messages are insulting her cleanliness and health protection abilities.^11^^,^^14^ A 2020 Bill & Melinda Gates Foundation review of malaria and gender documented evidence suggesting that employing women as spray operators and community mobilizers can help to address gender barriers and increase IRS acceptance.^15^ The 2018 study in Kenya and India reported that participants perceived women to have a strong understanding of their communities, thereby making them more likely to implement vector control programs that are accepted.^2^ The PMI AIRS and VectorLink projects have also found through implementation that female spray operators are strong communicators and effective in achieving high IRS acceptance.

### Recommendations

To accelerate and sustain gains in women’s involvement in vector control, country programs should work closely with national, regional, district, and local leaders to demonstrate the importance of hiring women in vector control—including leadership positions—and the impact on female economic empowerment, community well-being, and success of vector control programs. In addition to government actors, community stakeholders, such as community-based and civil society organizations, religious leaders, village leaders, community health workers, women’s groups, and youth groups, can play a role in maintaining and expanding inclusivity in the vector control workforce, including through addressing social norms. Sociocultural norms play a decisive role in enabling or hindering women’s involvement in vector control. Such norms vary by country and by factors, such as subnational geography, ethnic or religious group, or education level, among others. Understanding the nuanced sociocultural beliefs and norms in a given context—including through conducting additional research—is key to informing policies or programs that can appropriately respond to them. Finally, these efforts depend on equipping stakeholders with information and communication tactics to advocate for female engagement in vector control within their communities so that, ultimately, there is collective ownership and action advancing the goal of gender equality in malaria programming.

Sociocultural norms play a decisive role in enabling or hindering women’s involvement in vector control.

## Notes

**Peer Reviewed**

**First published online:** May 18, 2023.
